# Los costes indirectos de la cefalea tensional. Una revisión sistemática de la literatura

**DOI:** 10.1016/j.aprim.2021.102238

**Published:** 2022-01-22

**Authors:** Juan Ernesto del Llano Señarís, Nuno Nunes Correia, Laura Georgina Logusso, María Errea Rodríguez, Carlos Bringas Roldán

**Affiliations:** a Fundación Gaspar Casal, Madrid, España; b Sanofi Portugal, Porto Salvo, Portugal; c Sanofi España, Barcelona, España; d Investigadora independiente, Pamplona, España

**Keywords:** Cefalea tensional, Costes indirectos, Revisión sistemática de la literatura, Tension-type headache, Indirect costs, Systematic literature review

## Abstract

**Objetivo:**

El objetivo de este trabajo fue revisar sistemáticamente la literatura publicada con relación a los costes indirectos estimados asociados al TTH.

**Diseño:**

Esta revisión sistemática siguió la declaración de elementos de informes preferidos para revisiones sistemáticas y metaanálisis (PRISMA).

**Fuentes de datos:**

La revisión se realizó en dos bases de datos principales, PubMed y EconLit, y fue completada con la búsqueda de literatura gris.

**Selección de estudios:**

El criterio básico para la inclusión de estudios fue que presentaran al menos una medida de costes indirectos específicos del TTH.

**Extracción de datos:**

Se seleccionaron finalmente 12 estudios para la extracción de la información. De todos los artículos seleccionados se sintetizaron las características del diseño del estudio, los tipos de coste incluidos, así como el instrumento de medida, y los resultados principales.

**Resultados:**

La búsqueda arrojó en total 568 estudios. Se encontró heterogeneidad en los diseños y muestras/poblaciones de los estudios incluidos. Sólo dos estudios estimaron costes directos e indirectos para el TTH. Entre los resultados más destacables, encontramos un impacto moderado estimado de la discapacidad por TTH (entre 0,037 y 0,15 por persona, 0,06-0,09% para la población). Las pérdidas de productividad y eficiencia se observaron y fueron muy heterogéneas. La disposición a pagar por un tratamiento efectivo oscilaría entre $1,32 y $9,20 mensuales. La calidad de vida es baja, entre 28,2 y 28,4 puntos sobre 100, y la calidad de vida relacionada con la salud, parece mejorar significativamente con un tratamiento.

**Conclusiones:**

A pesar de la elevada heterogeneidad de los resultados, podemos concluir que la cefalea tensional se caracteriza por un impacto moderado en la discapacidad, en la productividad y eficiencia en el trabajo o la escuela, y en la calidad de vida de quien la sufre.

## Abstract (en)

**Objective:**

The objective of this work was to systematically review the published literature in relation to the estimated indirect costs associated with TTH.

**Design:**

This systematic review followed the Preferred Reporting Items Statement for Systematic Reviews and Meta-analyzes (PRISMA).

**Data sources:**

The review was performed in two main databases, PubMed and EconLit, and was completed with the gray literature search.

**Study selection:**

The basic criterion for the inclusion of studies was that they present at least one measure of indirect costs specific to TTH.

**Data extraction:**

12 studies were finally selected for information extraction. Of all the selected articles, the characteristics of the study design, the types of costs included, as well as the measurement instrument, and the main results were synthesized.

**Results:**

The search yielded a total of 568 studies. Heterogeneity was found in the designs and samples/populations of the included studies. Only two studies estimated direct and indirect costs for TTH. Among the most notable results, we find an estimated moderate impact of disability due to TTH (between 0.037 and 0.15 per person, 0.06–0.09% for the population). Productivity and efficiency losses were observed and were very heterogeneous. The willingness to pay for effective treatment would range from $1.32 to $9.20 per month. Quality of life is low, between 28.2 and 28.4 points out of 100, and health-related quality of life seems to improve significantly with treatment.

**Conclusions:**

Despite the high heterogeneity of the results, we can conclude that tension headache is characterized by a moderate impact on disability, on productivity and efficiency at work or school, and on the quality of life of those who suffer it.

## Introducción

En 2016, de todas las causas de enfermedades registradas en el *Global Burden of Disease* (GBD), el dolor de cabeza de tipo tensional (TTH), fue el más frecuente, con un total de 1,89 mil millones de afectados^1^. Se sitúa así por encima de la migraña, que aparece en sexto lugar a pesar de tener asociada una mayor carga de la enfermedad que el TTH^1^. De acuerdo con un estudio recientemente publicado, en Europa estima una prevalencia global anual de un 38% de la población^2^, aumentando esta tasa de prevalencia hasta el 79% si consideramos cualquier tipo de dolor de cabeza^2^. Además, las tasas de prevalencia de ambos tipos de dolor de cabeza, se han duplicado desde 2011^3^. Casi todas las personas experimentan o experimentarán un dolor de cabeza en algún momento de sus vidas^4^. En España, según un estudio con datos retrospectivos los costes médicos directos atribuibles al dolor de cabeza (de los cuales la migraña fue responsable del 50% de las hospitalizaciones, y el TTH de 14,47%) ascendieron a más de 10 millones de € anuales^3^. Las mujeres se ven más afectadas por este tipo de dolor de cabeza que los hombres, tanto en atención primaria (un 71,9% de mujeres), como hospitalaria (69,6%)^3^.

El TTH se clasifica como un trastorno individual, neurológico y no transmisible^1^. La carga global que supone está documentada, así como los costes directos, los costes de la medicación, los costes de hospitalización y otros costes de atención ambulatoria, en diferentes revisiones^5^, ^6^. El impacto socioeconómico de el TTH es sustancial^7^.

La literatura sobre los costes indirectos de TTH se basa primordialmente en recopilar estimaciones del valor (monetario) de las pérdidas de productividad y/o eficiencia asociadas al absentismo laboral o presentismo^8^. Las pérdidas de productividad por TTH debidas a una menor eficiencia en el trabajo se han estudiado desde principios de 1990, pero este factor se ha omitido comúnmente en el cálculo de costes indirectos de enfermedades debido a dificultades para su cálculo^9^. Además, a pesar de que se ha establecido en la literatura una relación inversa entre utilidad y costes indirectos de la enfermedad^10^, no encontramos ninguna revisión de literatura sobre costes indirectos de TTH que incluya la calidad de vida relacionada con la salud (CVRS) como uno de los componentes de los costes (o ahorros) indirectos de la enfermedad. Esto es así a pesar de existir instrumentos validados, comúnmente utilizados y bien establecidos para la medición de la calidad de vida (CV) (como el EQ-5D^11^,) y de calidad de vida relacionada con la salud (CVRS) (como el SF-36^12^,). La información recogida con dichos instrumentos es fácilmente transformable en utilidades (que a su vez pueden ser convertidos en pérdidas o ganancias monetarias), a partir de tarifas disponibles para varios países^a^, incluido España^13^. A pesar de no estar exentos de limitaciones^14^, no usar las ganancias o pérdidas de CV y CVRS a la hora de estimar los costes indirectos asociados a esta o cualquier otra enfermedad significa infra estimar dichos costes.

El objetivo de este estudio fue revisar sistemáticamente la literatura y ofrecer un resumen de los resultados encontrados en la literatura publicada sobre las medidas y estimación de los costes indirectos de la cefalea tensional.

## Métodos

### Estrategia de búsqueda

Esta revisión sistemática siguió la declaración de elementos de informes preferidos para revisiones sistemáticas y metaanálisis (PRISMA)^15^. Se aplicó el método PICO/PECO para estructurar^16^ b y se combinaron las palabras clave usando términos *booleanos*. Nuestra estrategia de búsqueda (disponible en material suplementario), para PubMed y EconLit, fue la siguiente:

(«*headache*» OR «*tension headache*» OR «*tension type headache*») AND («*indirect costs*» OR «*friction costs*» OR «*economic burden*» OR «*disease burden*» OR «*productivity costs*» OR «*productivity losses*» OR «*absenteeism*» OR «*presenteeism*» OR «*quality-of-life*» OR «*QoL*» OR «*quality of life*» OR «OTC» OR «*over the counter*» OR «*medication misuse*» OR «*medication underuse*» OR «*untreated*»)

#### Criterios de inclusión y exclusión

**Figura 1:**
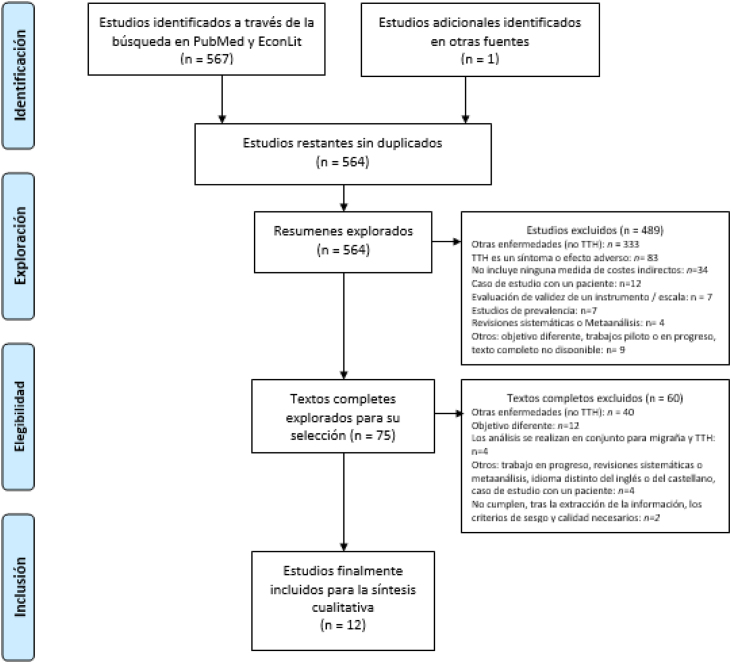
Diagrama de flujo PRISMA.

Limitamos los registros a cualquier artículo académico o literatura gris publicada y disponible en formato de texto completo, en inglés o castellano, seleccionando solo los que proporcionaran alguna información sobre los costes indirectos del TTH. Se admitieron tanto estudios descriptivos, como econométricos. Para estudios cualitativos, se contrastó que la fuente de la información fuera fiable, o que la población entrevistada tuviera conocimientos a nivel experto. No se filtró por grupos de edad, pero sí se extrajo la información con relación a la edad (según fueran presentados los resultados en el estudio) en la fase de revisión de textos completos, con el fin de asegurar incluir los resultados de la población en edad de trabajar. Todos los criterios de exclusión identificados aparecen detallados en el PRISMA (fig. 1).

### Extracción de datos

Todas las referencias identificadas fueron importadas a Zotero, software bibliográfico utilizado para la selección de estudios. La selección de estudios incluyó la exploración de títulos y resúmenes en una primera etapa, y textos completos en una segunda etapa. La búsqueda y selección fueron realizadas en enero de 2021 por dos investigadores, independientemente el uno del otro. Cualquier duda o desacuerdo entre los dos investigadores fue discutido con un tercer investigador. La metodología seguida para la extracción de datos fue revisada y aprobada por todos los autores. En ningún caso fue necesario ponerse en contacto con ninguno de los autores de los estudios incluidos en esta revisión para completar la falta de información relevante.

### Evaluación del riesgo de sesgo y evaluación de la calidad

Seguimos el método desarrollado por Parmar et al.^17^ para evaluar el riesgo de sesgo de nuestros registros incluidos.

Para ensayos de control aleatorizados, evaluamos el sesgo de selección mediante el análisis de la idoneidad de los métodos de muestreo (potencia, tamaño muestral, diseño ciego, aleatorización). Consideramos más fuertes aquellos estudios que proporcionaron información sobre las ganancias potenciales de un tratamiento con respecto a otros tratamientos, o a un grupo de control. Cuanto mayor es la distancia (en años) entre el período de tiempo analizado y el momento de publicación, mayor será el riesgo de sesgo de tiempo. Consideramos un mayor riesgo de medición en la variable de exposición para los tipos de tratamiento y resultados en salud auto reportados, no contrastados por personal clínico. En cada publicación se dio una puntuación, a cada dominio, de 1 para un riesgo bajo de sesgo, 2 para un riesgo moderado y 3 para un riesgo alto. A continuación, calculamos la calificación general de la siguiente manera: 1 (fuerte) se da si ninguno de sus dominios está clasificado como débil, 2 (moderado) si hasta dos dominios están clasificados como débiles, o 3 (débil) si tres o más dominios están clasificados como débiles.

**Figure:**
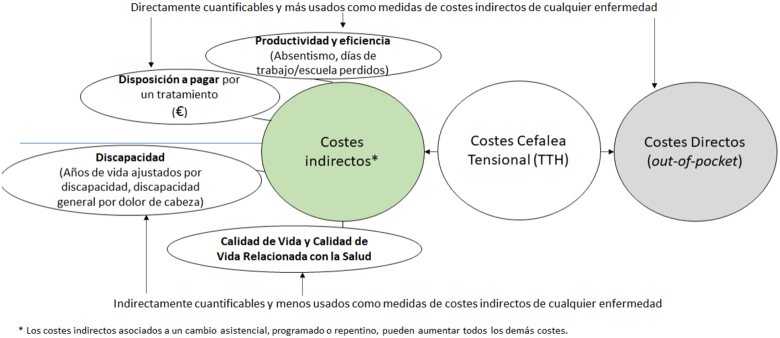
**Esquema general del estudio**.

## Resultados

Nuestra estrategia de búsqueda (en títulos y resúmenes) identificó 568 estudios potenciales de PubMed (557), EconLit^10^ y otras fuentes^1^ (un artículo fue recuperado manualmente dado el conocimiento del estudio y de su relevancia)^18^. Después de eliminar duplicados 564 resúmenes permanecieron para su revisión. Excluimos 489 resúmenes y seleccionamos 75 para la revisión completa del texto. Finalmente seleccionamos 14 artículos para la extracción de la información. Se optó por la exclusión de dos documentos tras la fase de extracción de datos, por no cumplir los criterios de Parmar et al.^17^ de calidad y sesgo.

En la figura 1 a continuación se presenta un diagrama de flujo PRISMA 2009 que representa el proceso de selección del estudio. Las razones de exclusión en las fases de exploración y elegibilidad aparecen detalladas en el PRISMA (fig. 1). Las listas de referencia de los informes de investigación principales las revisiones sistemáticas anteriormente publicadas con enfoque en el TTH se revisaron en un intento de identificar estudios adicionales. La extracción de datos y la evaluación detallada del riesgo de sesgo y de la calidad de los estudios están disponibles como material suplementario.

En la tabla 1 se resumen las medidas capturadas de costes indirectos y directos por los artículos seleccionados.

**Tabla 1:** Resumen de medidas encontradas de costes indirectos y directos en la literatura revisada

Medidas	Descripción	Referencias
**1-Costes Indirectos**		
*Discapacidad*		
Años de vida ajustados por discapacidad (DALY)	Suma de años de vida perdidos (YLL) a la mortalidad prematura y los años de vida vividos con discapacidad (YLD).	Stovner et al.^1^
Discapacidad atribuida al dolor de cabeza a nivel individual	Producto del tiempo en estado ictal y el peso de la discapacidad (DW) según GBD 2013.	Manandhar et al.^19^; Rastenyté et al.^21^.
*Henry Ford Headache Disability Inventory* (HDI)	Cuestionario con 25 items para describir la discapacidad de los pacientes con dolor de cabeza (de cualquier tipo)	Lee & Lee^22^.
Cuestionario HARDSHIP (*Headache-Attributed Restriction, Disability, Social Handicap and Impaired Participation*)	Cuestionario que incluye varios dominios de carga de la enfermedad, incluida la discapacidad por dolor de cabeza	Zebenigus et al.^20^.
*Pérdidas de productividad*		
Ausentismo	Pérdida auto reportada de días de trabajo; días libres debido a TTH o variable categórica (0 días, 1-7 días, 8-14 días, > 14 días durante el año anterior); porcentaje de ausencia en el año anterior	Karli et al.^23^; Linde et al.^6^; Rasmussen et al.^24^.
Pérdida de eficiencia	Número de días perdidos en en el trabajo/escuela o € anuales	Karli et al.^23^; Manandhar^19^.
Tiempo productivo perdido	Medido por el cuestionario HALT, como número de días perdidos en los últimos tres meses	Linde et al.^6^; Karli et al.^23^; Rastenyté et al.^21^; Zebenigus et al.^20^.
Reducción de la productividad (y su valor en €)	Estimada a partir de días en el trabajo en los cuales la cantidad realizada se redujo un 50%, cada uno de los días contados como un día completo perdido	Linde et al.^6^.
*Disposición a pagar*		
Disposición a pagar (WTP) por un tratamiento efectivo para el TTH	A los participantes se les preguntó cuánto estarían dispuestos a pagar por mes por un tratamiento eficaz, de modo que sus dolores de cabeza ya no les molestaran	Manadhar et al.^19^; Zebenigus et al.^20^.
*Calidad de Vida y CVRS*		
SF-12 HIT-6 WHQOL-8 SF-36	Cuestionarios en los que el participante ha de elegir los niveles de percepción con su estado de salud, que está compuesto por distintas dimensiones de salud física, mental o emocional	Espí-López et al.^25^; Lee & Lee^22^; Manandhar et al.^19^; Rastenyté et al.^21^; Zebenigus^20^.

**2-Costes Directos**		
Pagos *out-of-pocket*	Pagos que corren a cargo del paciente. Se agregaron en categorías, incluyendo: medicamentos agudos, medicamentos profilácticos, atención médica ambulatoria (incluidas las visitas a salas de emergencia y especializadas), hospitalización e investigaciones diagnósticas, y costes de proveedores de atención primaria.	Linde et al.^6^; Karli et al.^23^.

GBD: global burden of disease; HALT: Headache Attribute Lost Time; HIT-6: Headache Impact Test-6; SF-12: Short Form-12; DF-36: Short Form-36; WHOQOL: World Health Organization Quality of Life questionnaire.

La tabla 2 resume las características de los 12 estudios finalmente seleccionados. La tabla 3 expone la síntesis de resultados y los objetivos de cada estudio. Cuando el estudio proporcionó diferencias por género, o por tipo de tratamiento, se presentan los resultados desglosados para cada categoría.

**Tabla 2:** Descripción de los estudios incluidos

Autor (año) Nombre del estudio (referencia) ACRÓNIMO	Tipo de estudio, diseño	Tamaño muestral (muestra con TTH, %)	TTH diagnosticada / auto reportada	Rango de edad, media (desviación estándar o rango inter cuartil)	Tratamiento	Costes directos medidos (Sí/No)	Medida utilizada de costes indirectos
Espí-López (2016) ^25^	Ensayo de Control Aleatorizado	76 (100%)	Diagnóstico médico	18-65	1) Tratamiento de presión inhibidora suboccipital (SI); 2) Tratamiento de manipulación suboccipital (SM); 3) combinación de tratamientos SI y SM; 4) grupo de control	No	**CV** medida mediante el cuestionario SF-12
Gildir et al. (2019) ^26^	Ensayo aleatorizado, doble ciego, grupos paralelos	160 (100%)	Diagnóstico médico	20-50	Tratamiento de punción seca (DN). En el grupo de control se da punción seca simulada (SDN)	No	**CVRS** medida mediante el cuestionario SF-36
Stovner et al. (2018) ^1^	*Global Burden of Disease data* 2016	1,89 mil millones (63%)	Diagnóstico médico	5-95	No	No	La **carga de enfermedad** se estima en años de vida ajustados por discapacidad (DALY), que son la suma de los años de vida perdidos (YLL) hasta la mortalidad prematura y los años de vida vividos con discapacidad (YLD).
Karli et al. (2006) ^23^	Encuesta	159 episódica, 168 crónica (35%)	Diagnóstico médico	No especificado.	Sí, algunas personas recetadas por un médico, otras, automedicación (por ejemplo, naproxeno, paracetamol, metamizol, flurbiprofeno, ergotamina, ácido acetilsalicílico, diclofenaco, nimesulida, sumatripán, solmitriptán (el estudio no permite identificar cuáles fueron utilizados por pacientes con TTH)	No	**Pérdida de productividad** medida en días perdidos en el trabajo / escuela. Pérdida de eficiencia (medida en días) en el trabajo / escuela.
Lee & Lee (2019) ^22^	Ensayo de control aleatorizado	62 crónicos (100%)	Auto reportada a través de una encuesta	19-29	Programa de biorretroalimentación; Terapia manual; Estiramientos.	No	**CVRS** medida a través del cuestionario HIT-6, el cual incluye 6 preguntas en relación con el dolor, funcionamiento social, función de rol, funciones cognitivas, dolor psicológico, y actividad.
Linde et al. (2012) ^6^ *Eurolight project*	Encuesta	2.488 (29,5%)	Diagnóstico basado en los criterios de la clasificación internacional de trastornos del dolor de cabeza, 2 ª edición (ICI-ID-II)	18-65	Recuerdos de los encuestados sobre el uso de medicamentos agudos durante el mes anterior.	Sí. Estimaciones de costes directos que incluyen: pagos de bolsillo, más lo que pagó o reembolsó el gobierno o las compañías de seguros.	Eurostat. La **productividad perdida** se estimó a partir del número de días perdidos en el trabajo (ausentismo); la reducción de la productividad se estimó a partir de los días de trabajo en los que la cantidad de trabajo realizada se redujo en un ‡ 50%, contando cada día como un día completo perdido.
López-Bravo et al. (2020) ^28^	Encuesta	0 (0%) Se trata de una muestra de médicos facultativos que dan su opinión con respecto a la cefalea tensional	No aplica. Se trata de una muestra de médicos facultativos que dan su opinión con respecto a la atención de pacientes con cefalea.	No especificado	No aplicable	No	Se elaboró una encuesta (ver: https://ars.els-cdn.com/content/image/1-s2.0-S0213485320301079-mmc1.pdf) mediante preguntas de respuesta abierta y cerrada (única y múltiple) sobre la satisfacción de los pacientes con los cambios en la atención al paciente como resultado de la pandemia debido a la COVID-19.
Manandhar et al. (2015) ^19^ *HARDSHIP project*	Encuesta	863 (48,1%)	Diagnóstico médico	18-65	No	No	**WTP** por un tratamiento efectivo; **CV** medida a través del cuestionario WHOQOL-8; la **discapacidad** individual y de la población atribuible a el TTH se mide utilizando los pesos de discapacidad (DWs) publicados por el GBD2013. Ofrece estimación de **pérdidas de productividad** a través del tiempo perdido debido al TTH en los 3 meses anteriores utilizando el cuestionario HALT.
Rasmussen et al. (1992) ^24^	Encuesta	578 (549 en el último año, 74%)	Auto reportada, contrastada por un neurólogo	25-64	Sí. Para algunos de los pacientes. Preparados de ácido acetilsalicílico (59%), paracetamol (42%) los más frecuentes. Benzodiazepinas, neurolépticos, y antidepresivos fueron raramente utilizados.	No	**Pérdidas de productividad.** La tasa de ausentismo se mide como el % de ausencias en el año anterior. Días de descanso, medidos por intervalos (0 días, 1-7 días, 8-14 días, > 14 días).
Rastenyté et al. (2017) ^21^ Proyecto Eurolight	Encuesta	240	Diagnóstico basado en los criterios de la clasificación internacional de trastornos del dolor de cabeza, 2 ª edición (ICI-ID-II) y en otras preguntas que abordan varios componentes de la carga de la enfermedad. Las preguntas fueron realizadas por estudiantes de medicina capacitados.	18-65	No	No	**CV** medida a través del cuestionario WHOQOL-8; La **discapacidad** individual y de la población atribuible a el TTH se mide utilizando los pesos de discapacidad (DWs) publicados por el GBD2013. **Pérdidas de productividad:** Ofrece estimación del tiempo perdido debido al TTH utilizando el cuestionario HALT.
Sertel et al. (2017) ^27^	Ensayo de control aleatorizado	60 crónicos (100%)	Diagnóstico médico	18-55	Sí. Además de los medicamentos farmacológicos (que vienen dados), las pruebas de intervención para la terapia de conciencia corporal (BAT) y el ejercicio aeróbico.	No	El cuestionario. **SF-36** se utiliza como medida principal de **CV.** HIT se implementa además para conocer la **CVRS** a través de ítems que miden la gravedad del dolor, las habilidades laborales y durante el tiempo libre del paciente, la fatiga y las características cognitivas
Zebenigus et al. (2017) ^20^	Encuesta	493 (20,6%)	Auto reportada. Pregunta de detección para el dolor de cabeza en el último año	> = 18	No	No	Una versión culturalmente modificada del cuestionario HARDSHIP para medir la **discapacidad** de la población. **Pérdida de productividad** medida entre la población en edad de trabajar (18-65). Se mide también la **CV mediante el WHQoL** y la **WTP** por un tratamiento efectivo para el TTH.

CV: calidad de vida; CVRS: calidad de vida relacionada con la salud; DW: pesos de discapacidad; GBD: global burden of disease; HALT: Headache Attribute Lost Time; HIT-6: Headache Impact Test-6; SF-12: Short Form-12; SF-36: Short Form-36; TTH: cefalea tensional; WHOQOL: World Health Organization Quality of Life; WTP: disposición a pagar; YLD: años de vida perdidos debido a discapacidad; YLL: años de vida perdidos.

**Tabla 3:** Objetivos de los estudios y síntesis de resultados

Autor (año) Nombre del estudio (referencia) ACRÓNIMO	Objetivo del estudio	Síntesis de resultados
Espí-López (2016) ^25^	Evaluar la calidad de vida de los pacientes con TTH tratados durante cuatro semanas con diferentes técnicas de terapia manual	**CV.** Teniendo en cuenta su salud general, en general, los pacientes presentaron una salud relativamente deficiente antes del tratamiento, Todos los grupos mejoraron ligeramente después del tratamiento, aunque sin cambios importantes. 1) Para el grupo con tratamiento SI: Pre vs. Post = 0,24 (t = -1,11, p-valor = 0,27); Pre vs. Seguimiento = 0,51 (t = -2,51; p-valor = 0,02); 2). Para el grupo con tratamiento SM: Pre vs. Post = 0,03 (t = 0,140, p-valor = 0,89); Pre vs. Seguimiento = 0,12 (-0,46, p-valor = 0,64); 3) Para el grupo que recibió el tratamiento combinado: Pre vs. Post = 0,06 (t = -0,44, p-valor = 0,66); Pre vs. Seguimiento = 0,12 (t = -0,91, p-valor = 0,37); 4) Para el grupo de control: Pre vs. Post = 0,36 (t = -1,97; p-valor = 0,06); Pre vs. Seguimiento = 0,18 (t = -1,09, 0,28).
Gildir et al. (2019) ^26^	Explorar la efectividad de la punción seca en los puntos gatillo en pacientes con TTH crónica para reducir la intensidad, frecuencia y duración del dolor de cabeza y mejorar la calidad de vida relacionada con la salud (CVRS).	**CVRS.** Los efectos (diferencia de medias) encontrados según la diferencia de las subescalas SF-36 entre las puntuaciones de un mes de seguimiento y de pretratamiento fueron: 1) Grupo DN (N = 80): Funcionamiento físico: 14,8; Rol físico: 30,1; Dolor corporal: 36,7; Salud general: 22,9; Vitalidad: 25,6; Funcionamiento social: 23,6; Rol emocional: 36; Salud mental: 23,5. 2) Grupo SDN (N = 80): Funcionamiento físico: 1,1 Rol físico: 1,9; Dolor corporal: 3,3; Salud general: 4,8; Vitalidad: 1,9; Funcionamiento social: 3,6; Rol emocional: 11,3; Salud mental: 2,4. La diferencia entre las puntuaciones medias del SF-36 de los grupos DN y SDN en las dimensiones de salud general, funcionamiento social, rol emocional y salud mental, ya era significativa en el período previo al tratamiento. Al comparar las diferencias entre las puntuaciones SF-36 de DN vs. SDN, la diferencia en el pretratamiento no fue mayor de 10 puntos (a favor del grupo DN) en cualquier dimensión, mientras que, en el seguimiento, la diferencia entre los grupos es mucho mayor, y la diferencia entre los dos grupos es estadísticamente significativa para todas las dimensiones en este caso.
Stovner et al. (2018) ^1^	Proporcionar una descripción general de los métodos de carga mundial de morbilidad (GBD) aplicados al dolor de cabeza, presentar los resultados detallados de la actualización para 1990-2016 sobre la carga del dolor de cabeza en diferentes regiones del mundo y con las tendencias temporales, y discutir las implicaciones de estos resultados. tanto para futuras iteraciones de GBD como para políticas de salud en todo el mundo.	**Discapacidad.** La cefalea tensional causó 7,2 millones (4,6-10,5) de YLD en 2016, un aumento del 53,1% (47,5-58,4) de los 4,7 millones (3 0-7 0) YLD en 1990. Para la cefalea tensional, la tasa global de YLD estandarizada por edad por 100.000 habitantes fue 95,9 (61,5–140,0), un cambio de –0,2% (–2,5 a 1,9) de los valores de 1990. El porcentaje de todos los YLD debidos a cefalea tensional fue del 0,9% (0,7-1,2) en general: 1,0% (0,7-1,3) para las mujeres y 0,8% (0, 6–1,0) para hombres. Un pico en la prevalencia y la tasa de YLD se produjo entre los 35 y los 39 años. En ambos sexos, los porcentajes de todos los YLD fueron más altos en el grupo de 15 a 49 años (1,3% para la cefalea tensional), pero también fue alto en niños de 5 a 14 años (0,6%), en individuos de 50 a 69 años (0,7%) y en ancianos (0,3%). En mujeres de 15 a 49 años, la cefalea tensional causó 2,9 millones (95% IU 1 8–4 2) de Años de Vida con Discapacidad en 2016. Las proporciones de todos los **DALY** para ambos sexos fueron 0,3% (0,2 –0,4) para el dolor de cabeza de tipo tensional.
Karli et al. (2006) ^23^	Investigar la carga económica del dolor de cabeza sobre el paciente y la sociedad en los centros de atención terciaria para el dolor de cabeza en un país en desarrollo.	Los **costes directos** totales promedio por paciente para TTH fueron USD 93 (DE = 115,1, mediana = 39,1) en promedio para TTH episódica y 104,8 (DE = 122,3, mediana = 53,5) para TTH crónica. Esto se desglosa de la siguiente manera: costes de tratamiento farmacológico* = USD 63 para TTH episódica y 76,2 para TTH crónica. Costes de atención ambulatoria especializada*: 15,3 para ETTH y 15,7 para CTTH. Costes de evaluación diagnóstica = 13,9 para ETTH y 12,1 para CTTH. Gastos de hospitalización: 0,5 para ETTH y 0,1 para CTTH. Costos de proveedores de atención primaria (PCPs)*: 0,3 para ETTH y 0,6 para CTTH; **Costes indirectos**: Días de trabajo/escuela perdidos*: 0,9 días para ETTH, 1,2 días para CTTH. Días laborales/escolares ineficientes*: 5,3 para ETTH y 9 para CTTH.
Lee & Lee (2019) ^22^	Desarrollar un programa de tratamiento de TTH más eficaz centrado en mejorar la postura en pacientes en correlación con la postura de la cabeza hacia adelante (FHP) e investigar los efectos de varios métodos de tratamiento en los niveles de atención, estrés y calidad de vida en pacientes con TTH.	**Discapacidad & CV**. Se observaron efectos significativos de grupo por tiempo tanto para el *Henry Ford Headache Disability Inventory* (HDI), (F2,59 = 3,303; P < 0,017) como para las puntuaciones del HIT-6 (F2,59 = 3,409; P < 0,05). Dos semanas después de la intervención, se observaron disminuciones significativas en las puntuaciones del HDI en los grupos BF y ST (F2,59 = 8,073; P < 0,017), mientras que se observaron disminuciones significativas en HIT-6 en el grupo BF, en relación con los valores obtenidos para los grupos MT y ST (F2,59 = 12,542; P < 0,017). Los resultados sugieren que las disminuciones en el TTH mejoran la calidad de vida y el funcionamiento durante las actividades diarias, y que la terapia por Biofeedback es más eficaz que los otros dos métodos (terapia manual, y estiramientos) para reducir los síntomas de FHP y TTH.
Linde et al. (2012) ^6^ *Eurolight project*	Estimar la pérdida de recursos económicos a causa del dolor de cabeza en Europa	**Costes directos.** El coste medio anual por persona del TTH en todos los países fue de 303€ (IC 95% 230–376). Los **costes indirectos** representaron el 92% de los costes totales, y resultaron más atribuible a la reducción de la **productividad** (173€) que al **ausentismo** (105€). Entre los costes directos, la principal categoría contributiva fue la atención ambulatoria (11€), seguida de las investigaciones (6€), la hospitalización (5€) y la medicación aguda (3€). Los profilácticos contribuyeron muy poco. En Austria aparecieron algunos valores atípicos influyentes.
López-Bravo et al. (2020) ^28^	Conocer el impacto de la pandemia COVID-19 en las Unidades de Cefaleas en España y evaluar cómo imaginan el futuro de estas estructuras los neurólogos responsables.	En el corto y medio plazo, la mayoría de los encuestados prevén un aumento significativo en las listas de espera de primeras visitas, revisiones y procedimientos. Dada la prevalencia de la cefalea y el posible empeoramiento clínico de los pacientes, será fundamental garantizar la asistencia de aquellos con peor situación clínica o con cefalea de nueva aparición con datos de alarma. Las consultas presenciales de pacientes ambulatorios se han cancelado o sustituido por consultas telemáticas. Este cambio de asistencia sanitaria ha tenido un **impacto importante en las estructuras asistenciales de cefaleas** en España, donde el 96% de los neurólogos ha tenido limitaciones en su actividad habitual y el 75% se ha visto obligado a suprimir la actividad presencial. COVID-19: menos del 20% de las estructuras asistenciales encuestadas pudieron mantener con normalidad su consulta de técnicas (bloqueos anestésicos de nervios pericraneales o administración de toxina botulínica), que en su mayoría se demoraron o suspendieron. El incremento de la atención telemática parece haber sido bien recibido por los pacientes con cefalea, de forma que la gran mayoría reconoce los riesgos de exposición a la COVID-19 y están dispuestos a reducir los desplazamientos a los centros hospitalarios. Parece claro, por tanto, que la pandemia afianza la utilidad de la consulta telemática en pacientes con cefalea, pero ha identificado la necesidad de ampliar el uso de la telemedicina mediante sistemas virtuales para la comunicación con los pacientes y otros profesionales sanitarios. Por ello, deberán diseñarse nuevas estrategias para facilitar el cuidado de nuestros pacientes con cefalea, particularmente en aquellos que acuden regularmente para la realización de procedimientos o terapias parenterales. Dada la prevalencia de la cefalea y el posible empeoramiento clínico de los pacientes, será fundamental garantizar la asistencia de aquellos con peor situación clínica o con cefalea de nueva aparición con datos de alarma.
Manandhar et al. (2015) ^19^ *HARDSHIP project*	Informar las estimaciones de informes de políticas de salud de la carga atribuible a los trastornos primarios de cefalea en Nepal	La **WTP** se asoció positivamente tanto con la frecuencia como con la intensidad del dolor de cabeza (p < 0,001) y, por lo tanto, fue menor en TTH (WTP media [NPR / mes] = 1074, DE = 2673). **CV.** Las puntuaciones del WHOQOL se asociaron negativamente con la frecuencia e intensidad del dolor de cabeza (ambos p < 0,001); en consecuencia, entre los participantes con dolor de cabeza, la calidad de vida fue mejor en aquellos con TTH (puntuación media de WHQOOL - 8 = 28,2, SD = 3,7)**. Discapacidad.** Los valores correspondientes para TTH fueron 0,15 para DISper (discapacidad por persona con dolor de cabeza) y 0,06% para DISpop (discapacidad en la población). **Tiempo perdido atribuido a cefalea** (como número medio de días perdidos por persona en tres meses). Tiempo productivo perdido total = 2,2 días (DE = 4,7), mayor para las mujeres (2,3 días, DE = 4,3) que para los hombres (2,0 días, DE = 5,0): tiempo de trabajo remunerado perdido (0,9 días, DE = 3,1); tiempo de trabajo doméstico perdido (1,3 días, DE = 2,8); actividades de ocio social perdidas (0,2 días, DE = 0,9). Se encontraron diferencias significativas (p < 0,001) entre géneros para el tiempo de trabajo remunerado perdido (1,2 días para los hombres, 0,7 para las mujeres) y tiempo de trabajo doméstico perdido (0,8 días para los hombres, 1,6 para las mujeres), pero no por las actividades sociales y de ocio perdidas. 2,2 días de pérdida productiva total en 3 meses para los pacientes con TTH representaría el 2,3% del total de tiempo total productivo de una persona.
Rasmussen et al. (1992) ^24^	El objetivo fue estudiar el grado y tipo de utilización de los servicios de salud, los hábitos de medicación y la baja por enfermedad por cefalea primaria.	La tasa de **ausentismo** por cefalea tensional en el año anterior entre los pacientes que tenían un empleo remunerado fue del 12% (56/472), y de estos el 16% estuvo ausente durante más de 14 días. La duración más común de ausencia en este grupo fue de 15 a 30 días, pero dos mujeres con cefalea tensional tuvieron períodos muy largos de baja por enfermedad (> 180 días). La tasa de ausentismo por cefalea tensional en la población total fue del 9% (56/618). El 20% de los pacientes se ausentaron el año pasado debido a la CT. En promedio, los cálculos con respecto a la cefalea tensional son 5% ausentes durante cuatro días, 2% durante 11 días y 2% durante 20 días, lo que arroja un total de **820 días de trabajo perdidos por cada 1.000 ocupados en un año.**
Rastenyté et al. (2017) ^21^ Proyecto Eurolight	Informar la política en la Unión Europea (UE) evaluando el impacto del dolor de cabeza.	**CV:** Los participantes con TTH puntuaron 28,4 ± 6,0 (p = 0,126) en el WHOQoL. HY: HY se asoció con la **pérdida de productividad** para los pacientes con TTH: Días de trabajo remunerados perdidos = 0,5 para los hombres (SD = 2,9) y 0,5 para las mujeres (SD = 2,2). Días de trabajo domésticos perdidos = 1,2 para los hombres (DE = 3,0) y 1,6 para las mujeres (DE = 3,7). Días de trabajo perdidos medios totales = 1,7 para los hombres y 2,1 para las mujeres. Ocasiones sociales perdidas = 0,1 para los hombres (DE = 0,3) y 0,1 para las mujeres (DE = 0,4). Las **pérdidas de productividad** por TTH fueron mucho menores (1,7 para los hombres y 2,1 días para las mujeres según valoraciones medias en el HALT). **Discapacidad.** Para TTH, con un DW de 0,037, podemos calcular de manera similar una discapacidad media mucho menor por persona con el trastorno de 0.09% ([2,7 / 30] * [6,7 / 24] * 100 * 0,037). Para pMOH (DW 0,217 [29]), la discapacidad estimada (basada en números pequeños) es mucho más alta en 6,7% ([18,5 / 30] * [12,1 / 24] * 100 * 0,217).
Sertel et al. (2017) ^27^	Investigar el efecto de la terapia de conciencia corporal (BAT) y los ejercicios aeróbicos sobre el dolor y la calidad de vida en pacientes con CT.	En nuestro estudio, si bien se observó que ambos métodos de tratamiento eran más exitosos en comparación con el grupo de control para disminuir la frecuencia del dolor, la intensidad del dolor, la duración del dolor, la discapacidad relacionada con el dolor, el uso de medicamentos relacionados con el dolor y el aumento de la calidad de vida, se encontró que el programa de ejercicio aeróbico y BAT tienen efectos similares en términos de disminuir la frecuencia y duración del dolor y reducir la severidad de la discapacidad relacionada con el dolor, y BAT para ser más efectivo como tratamiento en las dimensiones de **CVRS** como rol-físico (pre = 41,39, SD = 12,3; post = 49,15, 10,48; valor de p 0,03), rol emocional (pre = 37,93; SD = 14,2; post = 47,40, SD = 13,17; p- valor = 0,00) y salud mental (pre = 37,49, SD = 9,68; post = 48,74, SD = 7,88; p = 0,02), mientras que la terapia de ejercicio aeróbico fue más eficaz como tratamiento para mejorar la percepción corporal (pre = 37,65, SD = 5,71; post = 46,15, SD = 7,37; valor p = 0,00) y la función social (pre = 36,32, SD = 6,24; post = 44.16, SD = 9.69; p = 0,01) dimensiones del instrumento SF-36.
Zebenigus et al. (2017) ^20^	Informar la política nacional de salud, mediante la presentación de datos de una encuesta sobre las cargas atribuibles al dolor de cabeza en Etiopía.	La **discapacidad** de la población fue del 0,06%. La discapacidad por TTH fue mucho menor que otros dolores de cabeza. Las personas con TTH crónica perdieron relativamente poco **tiempo productivo** (≤ 2.0%, diluido al 0,41% entre la población de 18 a 65 años) en comparación con otros dolores de cabeza. En promedio, en los tres meses anteriores, perdieron 1,3 (DE = 3,0) días de trabajo remunerado, 1,1 días (DE = 3,4) del trabajo doméstico y 0,2 días (DE = 0, 5) del ocio (DE = 1,74) [mediana = 0,79]. **CV.** 29,3 (DE = 3,0), p < 0,0001. Las pérdidas de tiempo productivo por TTH, aunque menores (2,0% y 1,2%, respectivamente), superaron sustancialmente la discapacidad estimada del 0,27%. Esto podría explicarse por las distribuciones sesgadas, y una minoría con relativamente alta discapacitada con frecuentes dolores de cabeza (alternativamente, sugiere que el DW de 0,037 atribuido a TTH en GBD2013 es demasiado bajo). La **WTP** para una atención médica eficaz para el dolor de cabeza. USD 1,32 por mes para TTH (DE = 1,74), lo que representa el 3,9% de la media de ingresos (DE = 6,2, mediana = 2,5).

BF: biofeedback; CTTH: cefalea tensional crónica; CV: calidad de vida; CVRS: calidad de vida relacionada con la salud; DE: desviación estándar; DN: punción seca; DW: pesos de discapacidad; ETTH: cefalea tensional episódica; GBD: global burden of disease; HALT: Headache Attribute Lost Time; HIT-6: Headache Impact Test-6; SDN: punción seca simulada; SF-12: Short Form-12; SF-36: Short Form-36; ST: estiramientos; TTH: cefalea tensional; WHOQOL: World Health Organization Quality of Life; WTP: disposición a pagar; YLD: años de vida perdidos debido a discapacidad; YLL: años de vida perdidos.* Diferencia estadísticamente significativa.

De acuerdo con las medidas de discapacidad, según el GBD^1^, el TTH causó 7,2 millones de YLD en 2016. Manandhar et al.^19^ encontraron pesos de discapacidad para el TTH de 0,15 por persona y 0,06% para la población (similar a los hallazgos en Zebenigus et al.^20^), mientras que Rastenytė et al.^21^ encontraron DWs de 0,037 por persona y 0,09% para la población. Además, Lee et al.^22^ utilizaron el Henry Ford *Headache Disability Inventory* (HDI) y encontraron mejoras, de efecto moderado, en los niveles de discapacidad en todos los grupos tras las intervenciones de terapia mediante *biofeedback* (BF) o *stretching*.

En lo relativo a las pérdidas de productividad y eficiencia en el trabajo, de acuerdo con los resultados de Karli et al.^23^ los pacientes con TTH perdieron, de media, en Turquía, 0,9 días de trabajo, y 1,2 si sufrían de TTH crónica. En el trabajo, los días ineficientes a causa de TTH fueron 5,3 y 9 para las TTH de tipo episódico y crónico, respectivamente. Encontraron, además, diferencias significativas entre los grupos de tratamiento y control para el absentismo y los días de trabajo/escuelas ineficientes (ambos fueron peores para los pacientes crónicos de TTH). Según Linde et al.^6^ los costes asociados al absentismo fueron de 105€ por persona al año, algo inferiores a los costes asociados al tiempo ineficiente en el trabajo, 173€ por persona/año. Además, según este mismo estudio los costes indirectos representaron el 92% de los costes totales. Manandhar et al.^19^ estimaron un total de 2,2 días perdidos, por persona y año, de los cuales 0,9 serían días perdidos de trabajo remunerado. Estos mismos autores estiman, para otro tipo de dolor de cabeza, que 20 días al año perdidos representan un 5,4% del tiempo productivo total de una persona al año, lo cual supone un 2,3% del tiempo productivo anual total de una persona. Rasmussen et al.^24^ estimaron tasas de absentismo equivalentes a un 12% de los pacientes con un empleo remunerado, un 9% de todos los pacientes, y un 20% cuando se trata de TTH crónica. En total, estimaron que el TTH supone al año un total de 820 días de trabajo perdidos por 1000 trabajadores. Rastenytė et al.^21^ encontraron pérdidas relacionadas con el tiempo ineficiente en el trabajo de 0,5 días (para hombres y mujeres por igual), de 1,2-1,6 días de trabajo doméstico, de 1,7-2,1 días medios totales perdidos, y 0,1 días de ocasiones sociales. A través del HALT, que mide la pérdida de productividad en los últimos tres meses, encontraron una media de 1,7 días para los hombres y 2,1 para las mujeres. Zebenigus et al.^20^, encontraron que las personas con TTH en Etiopía perdieron relativamente poco tiempo productivo en comparación con otros dolores de cabeza. En el promedio de los tres meses anteriores, perdieron 1,3 días de trabajo remunerado, 1,1 días de trabajo doméstico y 0,2 días de ocio. Esto se resume como pérdidas medias de $1,32 al mes para TTH (SD = 1,74), lo que representa el 3,9% de la media de ingresos, en media por persona.

Manandhar et al.^19^ encontraron, para Nepal, una disposición a pagar (WTP) de NPR 1.074 por mes (equivalente a $10,95 según la tasa de cambio NPR a USD ($) en 2014Zebenigus et al.^20^ encuentra que los pacientes con TTH estarían dispuestos a pagar por un tratamiento efectivo para esta enfermedad (en media, $1,32 /mes, desviación estándar = 1,74, mediana = 0,79).Consultado en: https://www.exchangerates.org.uk/NPR-USD-spot-exchange-rates-history-2014.html.

Espí-López et al.^25^ encontraron una mejora estadísticamente significativa en la CV de los pacientes con TTH en el seguimiento tras recibir un tratamiento de presión inhibidora suboccipital (SI) en comparación a la CV antes de recibir el tratamiento (diferencia de 0,51 puntos en el SF-12). No se encontraron diferencias significativas en la CV para los pacientes que recibieron el tratamiento de SI. En general, el estudio concluye que, aunque se observaron mejoras en todos los grupos, estas no fueron importantes. Lee et al.^22^, observaron una mejora en la calidad de vida (medida por el instrumento HIT- 6) de los pacientes cuando disminuye el TTH, especialmente en pacientes que recibieron un tratamiento con terapia (BF), aunque también con los otros tipos de terapia ofrecidos (terapia manual y estiramientos). Otros dos estudios, Manandhar et al. y Rastenytė et al.^19^, ^21^, compararon la CV de los participantes que sufren de diferentes tipos de dolores de cabeza, pero no controlan si los pacientes se encontraban o no bajo algún tratamiento. Ambos encontraron una CV (medida con el instrumento WHOQOL-8) pobre (de media 28,2 and 28,4, respectivamente) para los pacientes con TTH, a pesar de que es ligeramente mayor para TTH en comparación con otros dolores de cabeza. Ambos estudios encontraron diferencias entre la CV de los pacientes con TTH en comparación con la CV de pacientes sin dolor de cabeza, a favor de estos últimos. Esta diferencia no fue significativa según los resultados del estudio de Rastenytė et al.^21^, mientras que sí lo fue en el estudio de Manandhar et al.^19^. Este último estudio también encontró que los valores de CV en la muestra de pacientes con TTH eran ligeramente superiores a los valores de CV para los pacientes con migraña. Zebenigus et al.^20^ estudiaron la CV para ofrecer estimaciones de la discapacidad y otro tipo de cargas asociadas al dolor de cabeza, incluyendo TTH y otros tipos, encontrando un valor medio de 29,3 puntos en el WHQOoL para los pacientes con TTH.

Gildir et al.^26^ encontraron mejoras en todas las dimensiones del SF-36 a favor de los pacientes que recibieron el tratamiento nuevo («*dry needling*» [DN]) en comparación con el grupo de control (punción seca simulada [SDN], placebo). Además, el estudio controla por las puntuaciones en el SF-36 antes de recibir el tratamiento, y observan que, aunque había una diferencia de menos de 10 puntos antes de recibir el tratamiento, y, mucho mayor entre los grupos, después del tratamiento, con diferencias significativas en todas las dimensiones, las mayores encontradas en dolor corporal (diferencia de 34 puntos) y en el rol emocional (37 puntos). Sertel et al.^27^ utilizan también como medida de CVRS el SF-36, y comparan una terapia de concienciación de la enfermedad (BAT) y una terapia de ejercicio aeróbico. Este estudio encuentra que la terapia BAT es más efectiva que la terapia de ejercicio aeróbico para mejorar las dimensiones de rol físico (mejora de 7,76 puntos entre las puntuaciones antes y después del tratamiento), emocional (9,47 puntos de mejora) y la salud mental (4,42 puntos de mejora). Sin embargo, el ejercicio aeróbico es más eficaz de cara a mejorar las dimensiones relativas a la percepción corporal (4,49 puntos) y el funcionamiento social (7,65 puntos).

De acuerdo con Linde et al.^6^ entre los costes directos de ocho países de la Unión Europea, la categoría contributiva más importante era la atención ambulatoria (media por paciente y año, 11€), seguida de las investigaciones (6€), la hospitalización (5€) y los medicamentos para enfermedades agudas (3€). Los profilácticos contribuyeron muy poco. Cabe destacar que algunos países se caracterizaron por observarse valores atípicos, como es el caso de Austria. El estudio de Karli et al.^23^ donde analizaron datos de Turquía, los costes directos totales por paciente para el TTH fueron de $93 para el paciente con cefalea tensional episódica (ETTH) y de $104,8 para el paciente con cefalea tensional crónica (CTTH). Este desagregado de la siguiente manera: Costes de tratamiento de medicamentos, $63 para ETTH y $76,2 para CTTH; Costes de atención ambulatoria especializada, $15,3 para ETTH y $15,7 para CTTH; Diagnóstico Costes de trabajo, $13,9 para ETTH y $12,1 para CTTH; Gastos de hospitalización: $0,5 para ETTH y $0,1 para CTTH; Costes de los proveedores de atención primaria, $0,3 para ETTH y $0,6 para CTTH.

Finalmente, en esta revisión incluimos un estudio, por López-Bravo et al.^28^. Según resultados de este estudio, el 96% de los neurólogos ha tenido limitaciones en su actividad habitual y el 75% se ha visto obligado a suprimir la actividad presencial. Durante la primera oleada de la COVID-19 menos del 20% de las estructuras asistenciales encuestadas pudieron mantener con normalidad su consulta de técnicas (bloqueos anestésicos de nervios peri-craneales o administración de toxina botulínica), que en su mayoría se demoraron o suspendieron.

Los resultados con relación al análisis del sesgo y calidad de los estudios se muestran en la tabla 4.

**Tabla 4:** Análisis del sesgo y calidad de los estudios incluidos

Autor (año) Nombre del estudio (referencia) ACRÓNIMO	Sesgo de selección	Falacia ecológica	Sesgo de confusión	Sesgo de información	Sesgo temporal	Error de medida en la variable exposición	Error de medida en los resultados de salud	Evaluación general de la calidad y el riesgo de sesgo
Espí-López (2016) ^25^	Moderado	Moderado	Débil	Fuerte	Fuerte	Fuerte	Fuerte	Moderado
Gildir et al. (2019) ^26^	Moderado	Fuerte	Moderado	Fuerte	Fuerte	Moderado	Fuerte	Moderado
Stovner et al. (2018) ^1^	Moderado	Fuerte	Moderado	Fuerte	Fuerte	Fuerte	Fuerte	Fuerte
Karli et al. (2006) ^23^	Fuerte	Fuerte	Moderado	Fuerte	Moderado	Débil	Fuerte	Moderado
Lee & Lee (2019) ^22^	Moderado	Fuerte	Moderado	Moderado	Débil	Fuerte	Moderado	Moderado
Linde et al. (2012) ^6^ *Eurolight project*	Moderado	Fuerte	Moderado	Fuerte	Fuerte	Moderado	Moderado	Fuerte
López-Bravo et al. (2020) ^28^	Débil	Moderado	Moderado	Fuerte	Fuerte	Fuerte	Fuerte	Moderado
Manandhar et al. (2015) ^19^ *HARDSHIP project*	Fuerte	Fuerte	Moderado	Moderado	Fuerte	Fuerte	Fuerte	Fuerte
Rasmussen et al. (1992) ^24^	Fuerte	Fuerte	Moderado	Fuerte	Débil	Moderado	Moderado	Moderado
Rastenyté et al. (2017) ^21^ Proyecto Eurolight	Fuerte	Moderado	Moderado	Fuerte	Fuerte	Moderado	Moderado	Moderado
Sertel et al. (2017) ^27^	Moderado	Fuerte	Moderado	Fuerte	Fuerte	Moderado	Moderado	Fuerte
Zebenigus et al. (2017) ^20^	Fuerte	Fuerte	Moderado	Fuerte	Fuerte	Fuerte	Moderado	Fuerte

## Discusión

### Principales hallazgos

Entre los 12 documentos seleccionados, encontramos siete documentos en los que los participantes recibieron algún tratamiento^6^, ^22^, ^23^, ^24^, ^25^, ^26^, ^27^. Todos los documentos seleccionados incluyeron alguna medida de los costes indirectos del TTH, y dos incluyeron, además, medidas de costes directos del TTH^6^, ^23^.

En términos de discapacidad fue estudiada en cinco de los estudios incluidos^1^, ^19^, ^20^, ^21^, ^22^, encontrando un aumento significativo de los YLD del 53,1% desde 1990, con el pico de prevalencia para la población de 35 a 39 años, y una proporción de 0,3% (0,2-0,4) DALY para ambos sexos. Los resultados encontrados para discapacidad se muestran consistentes entre sí, presentando hallazgos similares. Además un estudio^22^ detalla cómo las terapias de ejercicio físico, tipo BF o estiramientos, pueden reducir de forma moderada los niveles de discapacidad. Seis estudios analizaron las pérdidas de productividad y eficiencia en el trabajo asociadas al TTH^6^, ^19^, ^20^, ^21^, ^23^, ^24^, pero en este caso los resultados fueron más difíciles de resumir en un intervalo o rango de valores estimados, dada la elevada heterogeneidad en los resultados encontrada, que puede ser explicada por la propia heterogeneidad en las medidas utilizadas. Dos artículos estudiaron la WTP por un tratamiento efectivo para el TTH. La disposición a pagar es inferior, pero muy similar, para un tratamiento efectivo para el TTH que para un tratamiento efectivo para otros dolores de cabeza, como la migraña. Siete estudios presentaron medidas de CV o CVRS^19^, ^25^, ^26^, ^27^. La CV es baja, entre 28,2 y 28,4 puntos sobre 100, y la CVRS parece mejorar significativamente con un tratamiento. El estudio de López-Bravo et al.^28^, a pesar de no incluir estimación de costes indirectos del TTH, ofrecen datos interesantes asociados al cambio de asistencia sanitaria, que ha tenido un impacto importante en las estructuras asistenciales de cefaleas en España. De acuerdo con los costes directos del TTH, a pesar de las diferencias en las pruebas encontradas por estos dos documentos, debemos tener en cuenta que uno está reportando valores medios para ocho países, y sobre la base de los datos de 2008 y 2009^6^, mientras que el otro se basa en los datos de 2001-2002 de una encuesta epidemiológica basada en una muestra de conveniencia en un único país^23^.

### Limitaciones

Aunque dado el tema de estudio, PubMed puede ser la base de datos que más probabilidad tenga de capturar estudios relevantes para nuestra pregunta de investigación, la segunda base de datos consultada, EconLit, apenas encontró artículos. Utilizamos Google Scholar para la búsqueda manual de algunos artículos que pudieran ser relevantes de integrar en la revisión. Además, presentamos resultados de las medidas que podrían cuantificarse como costes indirectos, y no la cuantía de costes indirectos que éstas supondrían en sí. La mayor parte de estudios están basados en análisis puramente descriptivos, y en fuentes de datos de tipo transversal. Además, la gran mayoría de los artículos publicados revisados estaba publicado en inglés, y este artículo ha preferido ser publicado en el idioma castellano.

### Recomendaciones

Es necesaria más evidencia con respecto a estas medidas como componentes de los costes indirectos del TTH, así como métodos estandarizados para la conversión de cada una de estas medidas en costes indirectos cuantificables. Futuras investigaciones en pacientes con TTH deberían incluir todos los componentes de costes indirectos analizados en este estudio.

Además, dada la elevada carga de la enfermedad, discapacidad, y pérdidas de productividad y eficiencia en el trabajo encontradas, asociadas con el TTH, y a la existente disposición a pagar por un tratamiento efectivo, deberían de tenerse en cuenta, en futuras investigaciones, el efecto preocupante que un cambio asistencial repentino ha tenido sobre la población de este tipo de pacientes. Esto podría servir para prevenir posibles costes indirectos derivados de cualquier situación futura que pudiera tener como resultado un empeoramiento clínico de algunos pacientes con TTH, especialmente de aquellos más severos, por el mero hecho de un cambio asistencial, ya sea súbito como programado.

## Conclusión

Existe un efecto de esta enfermedad, no sólo sobre la productividad y la eficiencia, sino también sobre la discapacidad, la calidad de vida, la calidad de vida relacionada con la salud. Existe una disposición a pagar positiva y similar, aunque ligeramente inferior, a la observada para otros tipos de dolores de cabeza. Sin embargo, la evidencia es, todavía muy escasa como para poder establecer que exista una única dirección para la relación y la magnitud de los costes, especialmente los indirectos, de padecer TTH con estas variables.

## Financiación

El estudio ha contado con una beca no finalista de Sanofi Consumer Health care Iberia.

## Conflicto de intereses

Nuno Correia y Georgina Logusso son empleados de Sanofi y pueden tener opciones de acciones. Juan del Llano, Carlos Bringas y María Errea no declaran conflictos de interés.

## Footnotes

a La lista disponible de tarifas se puede consultar en: https://euroqol.org/eq-5d-instruments/eq-5d-5l-about/valuation-standard-value-sets/

b El marco de esta revisión sistemática de acuerdo con PICO fue: Población: personas diagnosticadas o que declaran sufrir de cefalea tensional, o que tuvieran buen conocimiento clínico de la enfermedad (por ejemplo, especialistas en la enfermedad, tratamiento de pacientes con TTH); Intervención: Tratamientos TTH de cualquier tipo; Comparación: Tratamiento versus no tratamiento del TTH y/o antes versus después del tratamiento (cuando estuviera disponible), o evolución de medidas de costes indirectos con el paso del tiempo; Resultados: efecto del tratamiento del TTH en el ahorro indirecto de costes del TTH.
